# Complete Genome Sequence of Mumps Virus Isolated from Karnataka State, India

**DOI:** 10.1128/genomeA.01429-16

**Published:** 2017-01-12

**Authors:** Sunil R. Vaidya, Chandrashekhar G. Raut, Deepika T. Chowdhury, Venkat S. Hamde

**Affiliations:** a National Institute of Virology, Indian Council of Medical Research, Pune, India; b National Institute of Virology Unit, Rajiv Gandhi Institute of Chest Diseases Premises, Bengaluru, India; c Department of Microbiology, Yogeshwari Mahavidyalaya Ambajogai affiliated to Dr. Babasaheb Ambedkar Marathwada University, Aurangabad, India

## Abstract

We report the first whole-genome sequence of mumps virus isolated from a two-year-old girl with bilateral parotitis from a Chikkahallivana village in the Davangere district of Karnataka State, India. The genome of the Davangere mumps isolate was 15,384 bp in length and identical to previously published mumps virus (MuV) genomes from India. BLAST results show 99.1% identity with previously sequenced genotype C viruses isolated from the states of Maharashtra, Tamil Nadu, and Uttar Pradesh.

## GENOME ANNOUNCEMENT

Mumps is mainly a childhood disease characterized by fever and swelling of the parotids, and it also spreads to different organs, i.e., genital organs, pancreas, thyroid, salivary glands, urinary tract, and central nervous system. Mumps-containing vaccines have not been included in India’s Universal Immunization Program. The Indian Academy of Pediatrics suggested inclusion of the first dose of mumps vaccine at nine months and a second dose at 16 to 24 months (1).

Mumps virus (MuV) is a member of the *Paramyxoviridae* family and *Rubulavirus* genus. MuV is a single-stranded negative-sense RNA genome with 15,384 nucleotides that encodes fusion (F), hemagglutinin-neuraminidase (HN), nucleoprotein (NP), phosphoprotein (V/P/I), matrix protein (M), large protein (L), and small hydrophobic (SH) proteins (2). A standard protocol to define MuV genotypes was proposed by the World Health Organization. Accordingly, MuV was distinguished into 12 genotypes (3).

Recently, seroprevalence studies, vaccine studies, outbreak investigations, virus isolation, and virus genotyping studies were reviewed from India (4). This review indicated that mumps is a significant public health problem in India but remains ignored due to the absence of a surveillance and documentation system. Genetic characterization of mumps virus is an effective tool to track the transmission of wild types. However, very few studies have documented a genetic characterization of mumps viruses. Circulation of mumps genotype C has been reported from Maharashtra, Karnataka, and Tamil Nadu states and circulation of mumps genotype G from Maharashtra and Punjab states (4).

An outbreak of mumps was investigated during January 2014 in Chikkahallivana village in the Davangere district of Karnataka State, India (5). During this investigation, four mumps viruses were isolated in Vero cells. A representative mumps isolate, obtained from a two-year-old girl with bilateral parotitis, was subjected to full-genome sequencing, as described previously (6). The genome of the Davangere mumps isolate was 15,384 bp in length and identical to the previously published MuV genomes. The genome contains seven genes encoding nine known proteins in the order NP-V/P/I-M-F-SH-HN-L. BLAST results show 99.1% identity with previously sequenced genotype C viruses from the states of Maharashtra, Tamil Nadu, and Uttar Pradesh. At the amino acid level, the SH gene shows 97.89% identity, and the HN gene shows 99.0% identity with Indian genotype C viruses. The SH gene is crucial for genotyping purposes, and the HN gene plays an important role in antigenicity and the immune response.

### Accession number(s).

The whole-genome sequence obtained from Karnataka State has been deposited in GenBank under the accession number KX953297 (MuVi/Davangere.IND/01.14[C]).
